# Mutual learning and reverse innovation–where next?

**DOI:** 10.1186/1744-8603-10-14

**Published:** 2014-03-28

**Authors:** Nigel Crisp

**Affiliations:** 1 All Party Parliamentary Group on Global Health, House of Lords, SW1A 0PW London, UK

**Keywords:** Mutual learning, Reverse innovation, Frugal innovation, Co-development, International partnerships, Global health

## Abstract

There is a clear and evident need for mutual learning in global health systems. It is increasingly recognized that innovation needs to be sourced globally and that we need to think in terms of co-development as ideas are developed and spread from richer to poorer countries and vice versa. The Globalization and Health journal’s ongoing thematic series, “Reverse innovation in global health systems: learning from low-income countries” illustrates how mutual learning and ideas about so-called "reverse innovation" or "frugal innovation" are being developed and utilized by researchers and practitioners around the world. The knowledge emerging from the series is already catalyzing change and challenging the status quo in global health. The path to truly “global innovation flow”, although not fully established, is now well under way. Mobilization of knowledge and resources through continuous communication and awareness raising can help sustain this movement. Global health learning laboratories, where partners can support each other in generating and sharing lessons, have the potential to construct solutions for the world. At the heart of this dialogue is a focus on creating practical local solutions and, simultaneously, drawing out the lessons for the whole world.

## Background

The need for mutual learning between people and health services around the world-and the place in this for what is frequently called *reverse* or *frugal innovation-*are becoming clearer year by year as we recognise the enormity of the changes that face us. Firstly, disease patterns are changing: with a growing epidemic of non-communicable diseases and new strains of drug resistant communicable diseases appearing. Secondly, demographic and economic changes mean that the ageing populations of western societies need more care whilst the people of the rapidly growing countries of the south and east are beginning to demand more and better services. Thirdly, advances in science and technology offer new possibilities of treatment but may also increase both costs and inequalities. Fourthly, health and health care are no longer the sole preserve of the traditional professions and health systems: novel jobs and roles and new for-profit and not-for-profit organisations and partnerships are springing up to seize opportunities and plug the gaps. These and other changes are having a dramatic impact and mean that new ways for promoting health and providing health care are required.

It is interesting to note that whilst many countries are seeking to create or expand universal health coverage for their people, others in Europe and the west are struggling to find ways to maintain it. The issues they are facing are the same: how to emphasise health promotion and disease prevention, how to involve other sectors in health, how to engage citizens and patients in their own health and health care, and how to do so in an affordable and sustainable way. The solutions are also likely to be very similar.

While there has been an understanding of the potential for *reverse* or *frugal* innovation in other sectors for some time, this conviction is only recently growing within the field of health. There are no blueprints or examples to follow and there is a need for learning, experimentation and the development of new strategies and new services. Whilst different countries are affected in different ways, there are nevertheless many commonalities. The world is becoming more and more inter-connected and in health, as in every other area, we are increasingly inter-dependent in managing risk and acquiring knowledge. There is an evident need for sharing learning globally and ultimately for co-development in the field of health.

The World Health Organization has a particular role in providing guidance and promulgating knowledge, and many other bodies also have a mission to do so, ranging from professional bodies to universities, research institutions and commercial companies. Much, but not all, of this is about developing standards and approaches in high-income countries and spreading them around the world. There is still relatively little reverse flow of ideas and approaches from lower to higher income countries and institutions.

Much more needs to be done to support this reverse flow for two major reasons. Firstly, we are in danger of missing out on the ideas and innovations of more than half the world. Secondly, and perhaps even more importantly, we are ignoring the extraordinary potential of learning from people and countries, which frequently don’t have an established infrastructure and are freer to experiment and innovate. People in these countries are working all too often with a blank slate and could, if we saw it in this way, be learning on behalf of us all.

## Discussion

The processes for supporting reverse or frugal innovation ave often been in the form of partnerships between institutions in the north and south and, increasingly, south to south partnerships. I started to collect examples of these innovations 7 years ago when I realised how much we, in the United Kingdom, could learn from people who don’t have our resources and, crucially, our baggage and vested interests. India, of course, has many examples of *Jugaad* innovation, where people use the assets to hand to create the solutions they need [1]. So too has Africa, and everywhere else where determined people seek to make improvements. Formal and informal routes for data collection and thinking are starting to develop, including through competitions, *Grand Challenges* and, of course, academic publications.

*Globalization and Health* is already making a very important contribution in the academic realm. The journal’s thematic series, ‘Reverse innovation in global health systems: learning from low-income countries’–has within a year–been able to bring out the dynamism and creativity of this field to illustrate how the concept of reverse innovation is being developed by researchers and practitioners in many different places and in many different ways. The guest editors of the thematic series, Shams Syed and Viva Dadwal, alongside journal editor Greg Martin, lay out the vision for “global innovation flow” through bi-directional partnerships and learning [2]. They argue that this global innovation flow has existed for centuries, with ideas from one culture and civilisation spreading and eventually influencing others. The key here is to recognise this phenomena, remove barriers and accelerate the flow to the benefit of us all. Their editorship of this Journal is attempting to do just this. A brief review of the series so far shows how the series authors are deepening and widening our understanding of the field and its potential.

There is, firstly, an interest in understanding what precisely can be learned from other environments and countries. In the article on learning from the Brazilian Community Health Worker Model in North Wales Christopher Johnson et al. are looking for help in transforming their own system. They write: “*The potential shift from the current reactive, curative services targeted to those with expressed need or demand, to proactive, holistic whole-household based health promotion could be a sustainable solution to escalating workforce costs, empower patients and ensure more appropriate access to relevant services”*[3].

Meanwhile Felicity AE Jones and colleagues set out to understand what can be gained by volunteers from the UK’s NHS working in low and middle income countries. They conclude that, “*More work is required to quantify the costs and benefits of volunteering within health partnerships for individuals and institutions, and the associated challenges and barriers. Despite these limitations our analysis suggests that there is a strong theoretical argument that the skills acquired through volunteering are transferable to service delivery within the NHS and that the benefits to individuals and institutions could be maximised when volunteering is formally embedded within continuing professional development processes*” [4].

Other contributors describe both what can be learned and how to do so. Michael Cotton and colleagues in discussing global surgical care set out their position very clearly: “*Surgical innovations from LMICs have been shown to have comparable outcomes at a fraction of the cost of tools used in high-income countries. These innovations have the potential to revolutionize global surgical care. Advocates should actively seek out these innovations, campaign for the financial gains from these innovations to benefit their originators and their countries, and find ways to develop and distribute them locally as well as globally”*[5]*.*

Patricia Dandonoli writes about “Open Innovation*”* as a new paradigm for global health saying that: “*Open innovation can take various forms, from crowd-sourcing to structured organizational alliances and strategic co-ventures … this approach requires new ways of working and innovative business models”*[6].

Jacqueline DePasse and Patrick T Lee take a very direct and practical approach in seeking to design a model for *reverse innovation* in health care. They write: “ … *there is currently no framework for describing the stages of reverse innovation or identifying opportunities to accelerate the development process. This paper combines the business concept of reverse innovation with diffusion of innovation theory to propose a model for reverse innovation as a way to innovate in health care. …. We then propose four sets of specific actions that forward-looking policymakers, entrepreneurs, health system leaders, and researchers may take to accelerate the movement of promising solutions through the reverse innovation pipeline*” [7].

Colin P Thunhurst, like DePasse and Lee, draws on ideas and theories from elsewhere to describe the way the field is developing. He argues that there is a coming together here of two paradigms from the developed and the developing world respectively: “ … *the shift from a medical paradigm to a more holistic paradigm which emphasises the social, economic and environmental origins of ill-health…. (meanwhile*) *a parallel shift was taking place in the cognate field of operational research/systems analysis (OR/SA) which was adding greatly to our ability to analyse and to identify key points of intervention in complex systems”*[8].

Rwandan Health Minister, Agnes Binagwaho, and her colleagues bring many of these ideas together. Rwanda has managed the most extraordinary development of its health service since the genocide of 20 years ago, introducing social health insurance and improving services and achieving major improvements in health and life expectancy. It has also explicitly set out to learn and document it’s learning with partners from abroad: “*Most recently, the Rwandan government adopted a national Health Sector Research Policy to guide work from clinical trials to operational and social science research. Research based on local needs has been an engine of improvement in the health sector, and has contributed to Rwanda’s likely achievement of the health-related Millennium Development goals”*[9]*.*

### Summary

Four years ago I called for a Movement: changes in professional education and increases in exchanges between high and low and middle-income countries to facilitate shared learning and facilitate the transformation that needs to take place in health [10]. All these things are still needed but awareness of these issues is now growing. There is a much clearer research agenda developing around the practicalities of mutual learning, *reverse* and *frugal* innovation. Now, I believe, it is time for mobilisation of knowledge and resources, and bringing a systematic and organised approach to the entire field.

This is about the need to be explicit about what can realistically be achieved and how this can be done: what is the framework for success? What are the barriers to progress? How can the reluctance of health planners and professionals in high-income countries to learn from other countries be overcome? How can the matching lack of self-confidence in their own creations amongst people in low-income countries be addressed?

We need to see, as *Globalization and Health* is doing so well, continuous communication and awareness raising, which supports the Movement for change. We need practical examples where these ideas are built into professional education, as envisaged by the Lancet Commission on the future of professional education [11]. We need to see better-organised exchanges between institutions and countries as is starting to happen in the UK [12]. It is also time to establish systematic ways to learn together across the world–and take advantage of the potential for doing so in low and middle-income countries where there is more scope for creativity and innovation and where there will be less resistance from institutions and vested interests. Rwanda is leading the way by setting out to create a learning health system and conducting what it calls “*disciplined experiments*”.

Rwanda is working alongside colleagues from Harvard to share and develop this learning. In doing so it is acting as what I would call a “Global Learning Laboratory”. We need other such Laboratories where partners can support each other in learning lessons for the whole world. They need not all be in low and middle-income countries, of course, or be examples of *reverse* or *frugal innovations.* There are plenty of very interesting innovations taking place in other countries, including my own. The key point, however, is that they should be about creating local solutions and, simultaneously, drawing out the lessons for the whole world. They should not be examples of international development–where the west teaches the rest–but of co-development where we all learn and grow together.

## Competing interests

The author declares he has no competing interests.

## Authors’ information

Nigel Crisp is an Independent member of the House of Lords where he co-chairs the All Party Parliamentary Group on Global Health. He is the former Chief Executive of the English National Health Service (NHS) and Permanent Secretary of the UK Department of Health. He is also the author of the book “Turning the World Upside Down–the Search for Global Health in the 21st century”.

## References

[B1] Radjou N Prabhu J Ahuja S Jugaad Innovation: Think Frugal, Be Flexible, Generate Breakthrough Growth 2012 San Francisco: Jossey-Bass

[B2] Syed S Dadwal V Martin G Reverse innovation in global health systems: towards global innovation flow Glob Heal 2013 9 36 10.1186/1744-8603-9-36 PMC3846449 23992598

[B3] Johnson CD Noyes J Haines A Thomas K Stockport C Ribas AN Harris M Learning from the Brazilian Community Health Worker Model in North Wales Glob Heal 2013 9 25 10.1186/1744-8603-9-25 PMC3681592 23764067

[B4] Jones FAE Knights DPH Sinclair WFE Baraitser P Do health partnerships with organisations in lower income countries benefit the UK partner? A review of the literature Glob Heal 2013 9 38 10.1186/1744-8603-9-38 PMC3766651 24001319

[B5] Cotton M Henry JA Hasek L Value innovation: an important aspect of global surgical care Glob Heal 2014 10 1 10.1186/1744-8603-10-1 PMC3892040 24393237

[B6] Dandonoli P Open innovation as a new pdigm for global collaborations in health Glob Heal 2013 9 41 10.1186/1744-8603-9-41 PMC3847159 24000780

[B7] DePasse JW Lee PT A model for 'reverse innovation' in health care Glob Heal 2013 9 40 10.1186/1744-8603-9-40 PMC3844499 24001367

[B8] Thunhurst CP Public health systems analysis - where the River Kabul meets the River Indus Glob Heal 2013 9 39 10.1186/1744-8603-9-39 PMC3766021 24119439

[B9] Binagwaho A Nutt CT Mutabazi V Karema C Nsanzimana S Gasana M Drobac PC Rich ML Uwaliraye P Nyemazi JP Murphy MR Wagner CM Makaka A Ruton H Mody GN Zurovcik DR Niconchuk JA Mugeni C Ngabo F de Ngirabega J Asiimwe A Farmer PE Shared learning in an interconnected world: innovations to advance global health equity Glob Heal 2013 9 37 10.1186/1744-8603-9-37 PMC3765795 24119388

[B10] Crisp N Turning the World Upside Down: the Search for Global Health in the 21st Century 2010 London: Royal Society of Medicine Press

[B11] Frenk J Chen L Bhutta ZA Cohen J Crisp N Evans T Fineberg H Garcia P Ke Y Kelley P Kistnasamy B Meleis A Naylor D Pablos-Mendez A Reddy S Scrimshaw S Sepulveda J Serwadda D Zurayk H Health Professionals for a New Century – transforming education to strengthen health systems in an interdependent world Lancet 2010 376 9756 1923 1958 10.1016/S0140-6736(10)61854-5 21112623

[B12] All Party Parliamentary Group on Global Health Improving Health at Home and Abroad–How Overseas Volunteering from the NHS Benefits the UK and the World 2013 [http://www.rcpsych.ac.uk/pdf/Improving%20Health%20at%20Home%20and%20Abroad%20-%20Final%20Report.pdf]

